# The Effects of Levofloxacin on Testis Tissue and Spermatogenesis in Rat 

**DOI:** 10.22074/cellj.2016.3994

**Published:** 2016-04-04

**Authors:** Ramesh Ahmadi, Mehdi Ahmadifar, Elham Safarpour, Nazila Vahidi-eyrisofla, Mehraneh Darab, Ali Mohammad Eini, AliReza Alizadeh

**Affiliations:** 1 Department of Biology, Qom Branch, Islamic Azad University, Qom, Iran; 2 Department of Biology, College of Science, University of Science and Culture, Tehran, Iran; 3 Department of Embryology, Reproductive Biomedicine Research Center, Royan Institute for Reproductive Biomedicine, ACECR, Tehran, Iran; 4 The Toronto Institute for Reproductive Medicine, (Repromed), Toronto, Canada; 5 Islamic Azad University, Damghan Branch, Damghan, Iran; 6 Young Researchers and Elites Club, Science and Research Branch, Islamic Azad University, Tehran, Iran; 7 Department of Animal Science, Saveh Branch, Islamic Azad University, Saveh, Iran

**Keywords:** Levofloxacin, Spermatogenesis, Rat

## Abstract

Levofloxacin is one of the Fluroquinoline antibiotic groups, which affect on controlling infections, especially in reproductive organs. It has therapeutic use in numerous countries, but little information exists on the effects of Levofloxacin on spermatogenesis when it is used for infectious treatment. The current study was designed to determine whether Levofloxacin influences testis tissue and spermatogenesis in rats. In this survey 50 male Wistar rats 6-8 weeks (250 ± 10 g) were used: normal salin as sham and control groups and 3 treatment groups (0.03, 0.06 and 0.08 mg Levofloxacin\kg body weight) during 60 days. The experimental groups were daily gavages. After 60 days, they were anesthetized with ether and testes were taken for histopathology studies, sperm parameters evaluation and several hormone concentrations. Although testosterone concentration was not affected by Levofloxacin levels, follicle stimulating hormone (FSH) and luteinizing hormone (LH) concentration significantly increased by Levofloxacin consumption in 0.03 and 0.06 mg Levofloxacin\kg body weight groups (P<0.01). Moreover, sperm concentration decreased linearly as Levofloxacin was increased (200, 192, 170, 128 and 75×10^6^ sperm for control, sham, 0.03, 0.06 and 0.08 mg Levofloxacin\kg body weight, respectively, P<0.05). Testis tissue cuts in experimental group when the amount dosage of Levofloxacin increased cells solidarity to the primary and secondary spermatogonia. Adding Levofloxacin linearly reduced spermatocyte cells and amount of all cells in semenifer pipes tube (P<0.05). Levofloxacin as an antibiotic has histopathology effects on the spermatocyte cells, especially in high dose. Therefore, it might reduce fertility in male that requires further studies.

One of the main problems in the world of today’s medicine is infertility effects. In other words, approximately 50% of infertility is related to problems in male. Multiple environmental factors are life style (nutrition, drugs, smoking and alcohol), diseases (systemic, genetic, and infectious) and antibiotics. Antibiotics are one of the main factors which involved in male infertility (1, 2). The research indicates that antibiotics have effects on semen as well as known side effects on sperm mechanism during treatment period (3, 4). Composition of Cotrimoxazole induces the reduction of number and movement of sperm (5). Sulfasalazine couse infertility (6) as well as sperm count reduction (7), oligospermia (8) and sperm immotility (9) and abnormal morphology (10).

Levofloxacin is one of the Fluroquinoline antibiotic groups, which have effects on controlling infections, especially in reproductive organs. It has therapeutic use in more than 100 countries, but little information exists on the impacts of Levofloxacin on spermatogenesis when it is used for infection treatment. Sometimes patients need to take antibiotics for long period for about 50-70 days, whereas spermatogenesis duration is 64 ± 8 and 48 ± 5 days in human and rat, respectively (11, 12). Researchers have shown that antibiotics, such as Amoxicillin, Erythromycin and Cotrimoxazol reduce the sperm concentration, too (13). Levofloxacin is so effective in treatment of chronic infectious diseases. In many sexually transmitted diseases infections of the urogenital tract and diseases such as tuberculosis and brucellosis need long term antibiotics in take for treatment that sometimes takes 10 to 50 days of treatment. However, to our knowledge, the question of whether using Levofloxacin affects spermatogenesis in male has not been addressed. The current study was designed to determine whether Levofloxacin influences testis tissue in rats.

This study received the approval of the Ethics Committee of Royan Institute. In this study, 50 male Wistar rats 6-8 weeks (250 ± 10 g) were used. Duration of the survey lasted 60 days. The rats were exposed to lightness and then darkness for 12 hours, respectively. The room temperature stood at 23-25˚C as its moisture was counted as 50-55%. The experimental groups are normal salin as sham and control group and 3 treatment groups (0.03, 0.06 and 0.08 mg Levofloxacin\kg body weight). That dose is in human but we mixed drug with distilled water with due consideration to the rat weight (2). After 60 days, rats were anesthetized. Blood was centrifuged (Spectrafuge, 16MLabnet IntUSA) at 14,000×g for 15 minutes at 10˚C to obtain serum. Follicle stimulating hormone (FSH), luteinizing hormone (LH) and testosterone were measured by using laboratory procedures and commercial kits. Hematoxyline-eosin (H&E) several slides were studied by optical microscope equipped with camera. In survey of spermatogenic cells (spermatogonia, primary spermatocyte, spermatid, sperm, and sertoli cells) compared the amount of each one of cells in experimental groups and control ones. The data generated from the study were analyzed by ANOVA by using the General Linear Model (GLM) procedure of SPSS. Differences between treatment means were tested by Duncan’s Multiple Range test.

In current study gavages of several doses 0.03 and 0.06 mg/kg Levofloxacin did not affect cells and full sperms. But destruction in tubules as well as spermatogonia and primary spermatocytes were altered by 0.08 mg/ml Levofloxacin (Fig.1A-E, P<0.05). Also sperms are not fully formed because of high dose of drug and there is accumulation of interstitial fluid in tubules. Although testosterone concentration was not affected by Levofloxacin, FSH and LH concentrations significantly increased by Levofloxacin consumption (Fig.2A-C). Sperm concentration decreased linearly as Levofloxacin was consumed (200, 192, 170, 128 and 75×10^6^ sperm for control, sham, 0.03, 0.06 and 0.08 mg Levofloxacin\kg body weight, respectively).

The previous research reveals that Ciprofloxacin causes the expected death of cells by activating caspase3 which can play an important role in sperm reduction (14-18), sperm motility, and the increase of sliced DNA in sperm, and varicocele (19). Another former research done previously shows the substantial effect of Moxifloxacin on the number and motility of sperm, and the process spermatogenesis. This effect was so that it could reduce the number and motility of sperms and normal morphology (20-23).

One of these factors is to stay healthy in germinal cells create a balance between the number of live cells and dead ones both of which play a role in spermatogenesis and the rate of sperm production. The amount of germinal sexual cells has reduced because of different types of dead cells such as apoptosis (24). In recent years, some of conducted studies on animal laboratories have show the role of chemicals, radiation, infection, virus, cod, freeze and removal of pituitary gland from body when apoptosis occurred and virus cell death in testis tissue (12, 25, 26).

**Fig.1:**
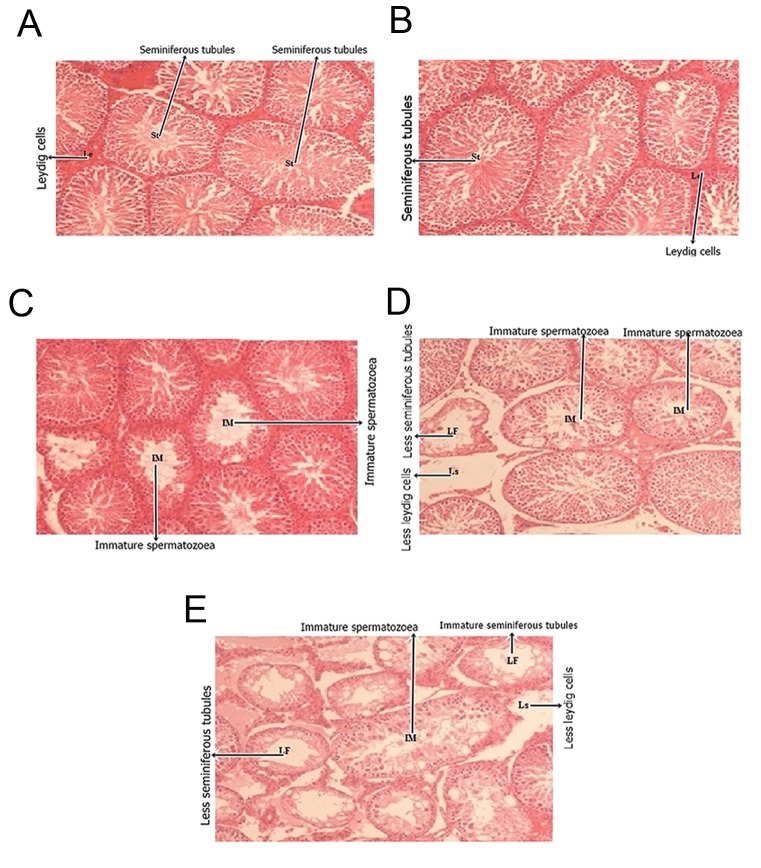
A. Microscopic image of the testis tissue of the control group, seminiferous tubules (St), and less Leydig cells (Ls) [Hematoxyline-eosin (H&E) stain ×200], B. Microscopic image of the testis tissue of the sham group, ST, Ls (H&E stain ×200), C. Microscopic image of the testis tissue of the 0.03 mg Levofloxacin/kg body weight, immature spermatozoea (IM), (H&E stain ×200), D. Microscopic image of the testis tissue of the 0.06 mg Levofloxacin/kg body weight, IM, less seminiferous tubules (LF), less Ls, (H&E stain ×200) and E. Microscopic image of the testis tissue of the 0.08 mg Levofloxacin/kg body weight, IM, LF, less Ls, (H&E stain ×200).

**Fig.2:**
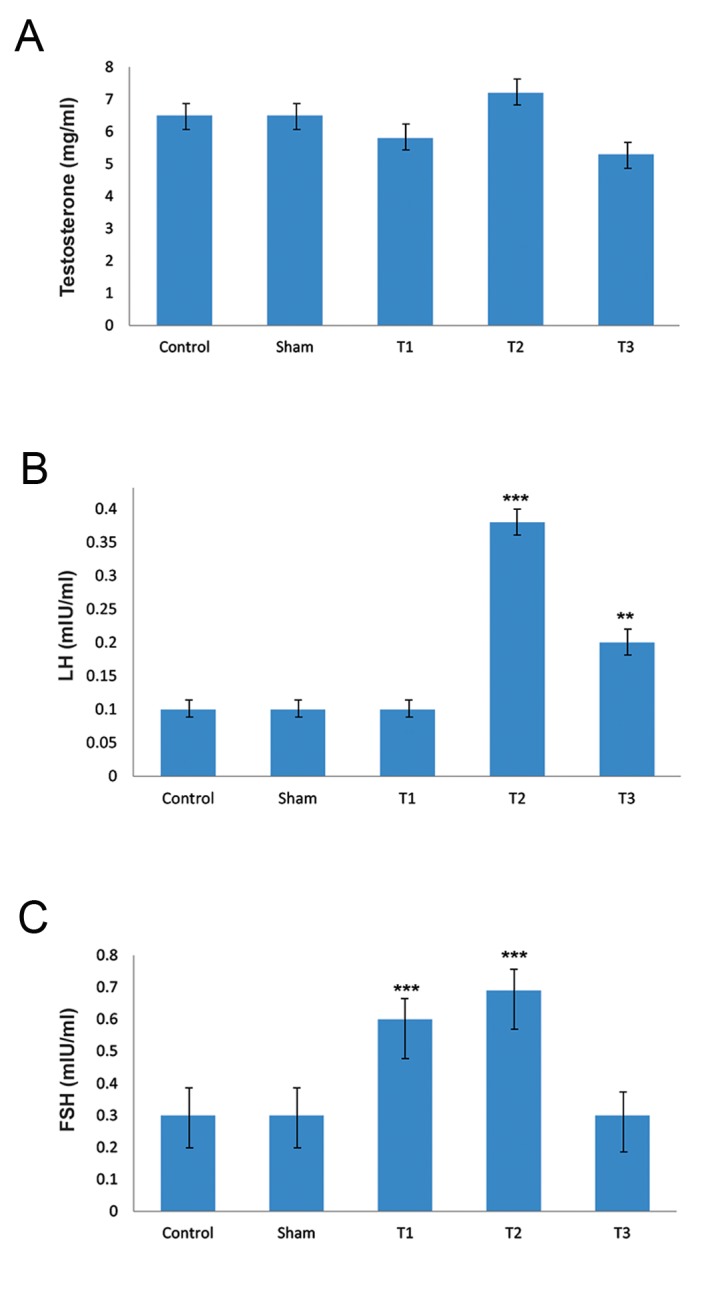
Testosterone concentration in experimental groups, B. LH concentration in experimental groups and C. FSH concentration in experimental groups. ***; P<0.001, **; P<0.01, T1; Group treatment by 0.03 mg/kg dosage, T2; Group treatment by 0.06 mg/kg dosage, T3; Group treatment by 0.08 mg/kg dosage , FSH; Follicle serum human and LH; Lutheal hormone.

It seems that several antibiotics might affect reproductive hormones by several pathways. Although the negative significant repercussion of Ciprofloxacin on FSH and LH concentration were reported in previous studies (14, 27), time-dependent and dose-dependent responses may explain the Levofloxacin effects on hormones. Indeed, schedule sampling in all studies with antibiotics was conducted at the completion of the experiment and the time of the maximum response has remained unclear in studies. Therefore, increased FSH and LH concentration in some experimental groups of these studies need more investigations.

Evaluation made on fluoroquinolones reveals these drugs have ripple effect on testis tissue and sperm parameters as well. It appears in artificial insemination (AI) process using Ciprofloxacins has priority to Gentamicin. The reason is that antibiotic in high dose is capable of destroying bacterial contamination of semen with less undesirable impacts on the functions of sperm. Further surveys are required so as to make an efficient comparison between fluoroquinolones and aminoglycosides. For evaluation of fluoroquinolones and aminoglycosides analogues parameters are applied. Moreover, it is proposed that further research on all drugs as part of aminoglycosides and fluoroqinlones be carried out on human beings. In a separatc survey conducted on Gentamicin’s toxicity on rate sperm, reduced caudal epididymal sperm reserves administration of Gentamicin. Ensuing Gentamicin administration sperm mobility decreased (28).

Sex hormones (LH and FSH) and spermatogenesis (sperm count, motility and viability) were significantly decreased in test group compared to those of controls (P<0.05). Ciprofloxacin has some adverse effects on sperm related variables in 28 day period. It appears that dose-depends response is important in this issue; because the testes cells in high dose groups try to compensate the destroyed cells by incensing FSH and LH concentration.

Altogether, the administration of therapeutic doses of Levofloxacin 0.08 mg/ml for 60 days can cause pathological changes such as atrophy. Seminiferous tubule and irreversible damages to the cells in the testis cause death to the spermatogonia cells and primary spermatocytes disrupt the normal cycle of spermatogenesis and caused hypo spermatogenesis and infertility in male rats. Thus, our results in high dose treatment support the theory that the use of some antibiotics such as Levofloxacin disturbs spermatogenesis, particularly Leydig cells, primary and secondary spermatocytes as well as Sertoli cells. Our results suggest that unlike other antibiotics this drug have dose-depends effects on all parameters of in spermatogenesis and have more histopathology effects.
